# The Impact on Family Functioning of Social Media Use by Depressed Adolescents: A Qualitative Analysis of the Family Options Study

**DOI:** 10.3389/fpsyt.2015.00131

**Published:** 2015-09-23

**Authors:** Andrew J. Lewis, Tess Knight, Galit Germanov, Michelle Lisa Benstead, Claire Ingrid Joseph, Lucinda Poole

**Affiliations:** ^1^School of Psychology, Deakin University, Burwood, VIC, Australia

**Keywords:** social media, adolescent mental health, depression, parenting, family based intervention

## Abstract

**Background:**

Adolescent depression is a prevalent mental health problem, which can have a major impact on family cohesion. In such circumstances, excessive use of the Internet by adolescents may exacerbate family conflict and lack of cohesion. The current study aims to explore these patterns within an intervention study for depressed adolescents.

**Method:**

The current study draws upon data collected from parents within the family options randomized controlled trial that examined family based interventions for adolescent depression (12–18 years old) in Melbourne, Australia (2012–2014). Inclusion in the trial required adolescents to meet diagnostic criteria for a major depressive disorder via the Structured Clinical Interview for DSM-IV Childhood Disorders. The transcripts of sessions were examined using qualitative thematic analysis. The transcribed sessions consisted of 56 h of recordings in total from 39 parents who took part in the interventions.

**Results:**

The thematic analysis explored parental perceptions of their adolescent’s use of social media (SM) and access to Internet content, focusing on the possible relationship between adolescent Internet use and the adolescent’s depressive disorder. Two overarching themes emerged as follows: the sense of loss of parental control over the family environment and parents’ perceived inability to protect their adolescent from material encountered on the Internet and social interactions via SM.

**Conclusion:**

Parents within the context of family based treatments felt that prolonged exposure to SM exposed their already vulnerable child to additional stressors and risks. The thematic analysis uncovered a sense of parental despair and lack of control, which is consistent with their perception of SM and the Internet as relentless and threatening to their parental authority and family cohesion.

## Introduction

There is a growing body of research focused on the complex relationship between adolescent depression and Internet and social media (SM) use (1–5), although the nature of this relationship remains unclear (1). Family factors and parental support are also associated with child and adolescent depression (6), and parental attitudes have also been found to be associated with a wide range of adolescent health behaviors (7, 8). However, very little is known about how family factors interact with SM use and adolescent depression.

In order to better understand how SM might influence family processes in the context of adolescent depression, the current study investigated parent’s views about SM use by their depressed adolescents who were 12–18 years old. The study employed a qualitative analysis based on transcripts derived from the family options (FO) study, which was a randomized controlled trial (RCT) of two family based interventions for treating adolescent depression, conducted in Greater Melbourne, Australia between 2012 and 2014 (9). This research was motivated initially by clinician observations that many parents within this trial were particularly concerned by their adolescent’s SM use. Parents in the treatment groups were frequently recounting incidents where their adolescents displayed clinically concerning interactions within online environments. Moreover, neither intervention in the clinical trial included advice or strategies that would assist parents to address their concerns about SM. The current analysis was undertaken with the long-term goal of developing effective therapeutic content to assist such families.

Social media use by children and adolescents is increasingly a significant feature of contemporary family life. In this study, we use the term SM to refer to the use of any website or digital device that facilitates social interactions (4), including SMS texting, Facebook, You Tube, Instagram, etc. The current generation of adolescents is the first to be raised predominately in a SM environment (1) and SM use is pervasive. For example, a study by the Australian Communications and Media Authority on young Australian’s use of SM that surveyed 819 children aged 8–17 reported that adolescents (10–15) used the Internet on average 6.3 days/week, for a period of approximately 3 h/day (16). This generation makes extensive use of SM to facilitate social interaction (17), perceiving the virtual world as an extension of the physical world (10) and considers online friendships to be valid and real. On the other hand, parents are often less knowledgeable and technologically competent than their adolescents in SM platforms (4, 5). This potentially compromises their ability to monitor and guide their adolescents’ social interactions or exposure to harmful content. One study suggested that parents felt that they had minimal control over what their children were exposed to over the Internet, whether it was harmful content or interactions, and that parents felt that their ability to protect their child was thereby compromised (5).

The risk–benefit profile of SM use in adolescence remains a matter of considerable debate. SM offers an adolescent a virtual space, which is independent of their parents. In such an environment, an adolescent needs to exercise their own judgment about safe practices (11). The anonymity of many forms of online communication can enable greater aggression and exposure to intimate or confronting information (12, 13). Cyberbullying, increased exposure to self-harm (1), privacy issues, and sexting (4) have been raised as concerning aspects of the online environment. At the same time, SM has great potential to facilitate learning, access to information and social connectedness.

The current study does not address the broader issue of SM use but is focused on a specific clinical group: adolescents with depression. Studies suggest a strong association between adolescent depression and excessive use of SM (1–3). For example, one study of six European countries found a positive association between heavy SM use (2 h or more per day) and internalizing disorders (depression and anxiety) with the relationship being more pronounced in younger adolescents (12, 13). Some researchers have used the term “Facebook Depression” to describe a phenomenon where adolescents spend extended periods of time on Facebook in the context of depressive symptoms (4). However, a recent systematic review of 43 studies examining the relationship between online communication, SM use and adolescent mental health concluded that evidence at this stage is inconclusive and insufficient to suggest a causal relationship (1).

The current study aims to examine the relationship between SM and Internet use within a context where adolescent depressive disorders have been diagnosed and families are attending a multi-group, family based treatment for depression. The study draws on transcripts of parent group therapy sessions and therefore presents the perspective of parents about their adolescent’s SM and Internet use. In addition, the current research aims to explore the impact SM and the Internet uses have on family cohesion and dynamics. The aim is to provide information that can be used to develop therapy and educational resources for parents and adolescents.

## Materials and Methods

### Study design

The current study draws upon data collected within the Family Options (FO) multicenter RCT that compared two family based interventions for adolescent depression (12–18 years old) in greater Melbourne, between 2012 and 2014. The two interventions consisted of fully manualized interventions known as the behavior exchange system training (BEST-MOOD) and parenting adolescents support training (PAST). Families of adolescents with depressive disorder were allocated to both BEST and PAST interventions and full inclusion and exclusion criteria for the trial are available in the published trial protocol (9). Both interventions consisted of eight sessions of weekly family group therapy, in which a group of parents attended the first four sessions, followed by four sessions that included the young person and, where relevant, their siblings. Previous studies of similar family based interventions have found improvements in parental mental health and family functioning (14, 15).

During assessment for entry into the RCT, adolescents were assessed for a unipolar depressive disorder (major depressive disorder or dysthymic disorder) using the mood disorders schedule of the Structured Clinical Interview for DSM-IV Childhood Disorders (KID-SCID). Adolescents presenting with bipolar disorder or any psychotic disorder were excluded.

### Participants

Participants in the current study were 39 parents whose adolescents were primarily recruited from the intake service of Eastern Health’s Child and Youth Mental Health Service (CYMHS), and some through community referrals. For the purpose of the current study, only the first four sessions in each intervention consisting of group sessions where only parents attended were transcribed and analyzed. The average age of parents in the sample was *M* = 47 years (SD = 5.55) and ranged from 32 to 65 years of age. In terms of sample characteristics, 20% of parents had obtained a post secondary school qualification (Undergraduate or Postgraduate Degree); 19% received some kind of government pension (sickness or disability); 40% were married and 33% were divorced and 15% described themselves as “single parents”; 55% of families earned <$A50 000 per annum. These sample characteristics suggest that, in an Australian context, these families are very slightly below average socio-economic status, but are demographically more or less typical of outer suburban Australian families.

All available recordings of therapy groups involving only parents were reviewed in order to obtain content relevant to the current analysis. The transcribed BEST and PAST interventions consisted of 56 h of recordings. All parents who attended these groups provided permission to be recorded and were included in the present analyses. Thirty-nine participants took part in the 7 intervention groups; 23 were mothers and 16 were fathers as presented in Table 1.

**Table 1:** **Therapy sessions (BEST and PAST) transcribed and frequency of social media content**.

Intervention	Participants	Relevant content – yes/no/bad recording (BR)
BEST, Term 4 2012	2 Couples and 1 mother	First session – BR
	3 Women	Second session – N
Location: Geelong	2 Men	Third session – Y
	Total: 5	Fourth session – N
PAST, Term 4 2012	2 Couples and 1 mother	First session – BR
	3 Women	Second session – Y
Location: Geelong	2 Men	Third session – BR
	Total: 5	Fourth session – BR
BEST, Term 1 2013	4 Couples	First session – Y
	4 Women	Second session – N
Location: Ringwood	4 Men	Third session – N
	Total: 8	Fourth session – N
PAST, Term 1 2013	2 Couples and 2 mothers	First session – Y
	4 Women	Second session – Y
Location: Ringwood	2 Men	Third session – N
	Total: 6	Fourth session – N
BEST, Term 2 2013	1 Couple and 1 father	First session – N
	1 Woman	Second session – Y
Location: Ringwood	2 Men	Third session – N
	Total: 3	Fourth session – Y
BEST, Term 1 2014	1 Couple and 2 mothers	First session – N
	3 Women	Second session – Y
Location: Ringwood	1 Men	Third session – N
	Total: 4	Fourth session – Y
PAST, Term 1 2014	3 Couples and 2 mothers	First session – Y
	5 Women	Second session – Y
Location: Ringwood	3 Men	Third session – Y
	Total: 8	Fourth session – N
Total Groups = 7 Hours per session = 2 h Total session hours = 56	Total number of participants = 39 (Mothers = 23, Fathers = 16)	Relevant content from BEST and PAST sessions = 12 (50%)

Relevant content to the research question was identified after the clinical trial had been completed and based on the current research question. Such content was discussed in 12 of the 28 recorded sessions. No relevant content was identified in 12 other sessions, and no transcripts were produced from 4 sessions that were of poor audio quality or were not recorded due to equipment failure. Table 1 provides detailed information on groups and session transcriptions from those groups.

The transcripts of four BEST and three PAST intervention groups were used for the purpose of this study. No differentiation was made between BEST and PAST transcripts, since neither intervention contained material about SM or Internet use, and there was no reason to assume the topic would be raised more or less in either interventions. This assumption proved correct when transcriptions were reviewed.

### Procedure

The study was approved by Deakin University Human Ethics Committee and all participants consented to audio recording of all group therapy sessions. All BEST and PAST interventions were recorded using *Livescribe* software. This software works in conjunction with a recording pen device to allow identification of speakers within an audio recording. These recordings were uploaded and stored in a secured network drive. The recordings from the seven interventions were transcribed by two authors on the paper (Galit Germanov, Claire Ingrid Joseph), accessing the files from a secure location. The complete transcript was forwarded for a preliminary review by Andrew J. Lewis and Tess Knight prior to conducting full thematic analysis by Galit Germanov and Tess Knight. Initial findings were cross-examined by Andrew J. Lewis and amended in line with discussions of its thematic meaning.

### Data analysis

Thematic analysis was employed, since it was particularly suitable for answering questions regarding people’s concerns about an event or phenomenon (18) and has been employed successfully by the current researchers to analyze similar clinical information previously (19). In this case, the focus was on parent’s perceptions and concerns regarding their adolescent’s use of SM and its impact on the adolescent’s mental health. Thematic analysis provides a complex account of the data, through the process of extracting common themes and drawing meaning from the accumulated evidence in a way that contributed to the understanding of the actual behavior or event (18).

In the current study, themes were extracted using the Braun and Clark’s (2006) thematic analysis model (20). The process consisted of identifying relevant content to the research question, coding the content according to its meaning, and extracting common categories/themes that provided a higher level of interpretation of the content, reflecting the underlying concerns and perceptions of the parents in the groups. The final stages of the analysis included the integration of the themes into a coherent story that shed light on the phenomenon investigated, using prior research to validate and support findings.

## Results

As presented in Table 1, 12 of the available 24 therapy sessions contained at least some content where parents discussed the theme of SM or Internet use by their adolescents. This rate of 50% indicates that this topic was a common one and it was typically raised as an issue of concern to parents. As noted above, neither therapy model (PAST or BEST) has any specific content that would elicit discussion of SM or the Internet so this high rate of discussion suggests that many parents in these groups raised the topic independently and frequently.

The thematic analysis suggested two overarching themes from the data with sub-themes for each: (a) the sense of loss of parental control over the family environment and (b) parents’ perceived inability to protect their adolescent. The overarching themes were in themselves inter-related since it appeared that the perceived loss of control led parents to believe that they were unable to protect their adolescent. Accordingly, sub-themes in one cluster often overlapped with themes from the other cluster illustrating a complex network of relationships between the themes.

### Parental sense of loss of control over the family environment

A consistent overarching theme that appeared was the parents’ sense of loss of control over the family environment, as a result of the penetration of Internet use and subsequently SM use into their home and lives. Parents’ sense of loss of control was attributed to three features of the Internet and online communication; accordingly, these were identified as the subordinate themes as follows: (a) the Internet allowed easy and discrete access to content and people, (b) virtual communication was relentless; it had no boundaries in space or time, and (c) children were natives to the virtual realm and therefore were often more technologically savvy than their parents.

#### Easy and Discrete Access to Content and People

Easy and discrete access to content and people caused parents to feel that they were no longer in control of who could come into contact with their children; accordingly, their abilities to respond to situations that may have influenced their child, or monitor their child’s behavior, were compromised. As one father described:

In our days, the phone rang, your dad would hear it, “your friend, is on the phone”, they knew who you’re on the phone to, how long for and whether you were affected by the conversation; … it was easy to be aware, but now they disappear into their room and you know physically where they are, but not mentally, or what they’re putting themselves to, or where they’ve been, or been subjected to.

The transition of the adolescents’ activities from the physical observable realm to the virtual discrete realm meant that parents were required to develop more explicit methods of monitoring that were perceived by their children as more intrusive and therefore created further conflict and tension between parents and children. As remarked by a mother:

It’s just another thing you have to monitor and they feel annoyed because they feel like they are being watched all the time; it just causes more friction at home

#### Virtual Communication is Relentless. It has no Boundaries of Space or Time

A prominent discussion across interventions revolved around the excessive use of the Internet and SM by adolescents, and their inability to disengage, as one mother commented:

They get home and they are like, it’s on the go, there’s no break from it! The communication is 24/7 …

The relentless nature of the Internet communication meant that family schedules and routines were constantly disrupted by events that happened on the Internet. Accordingly, parents were required to respond to fast changing situations that impacted their adolescent’s mood and behavior, which influenced the interactions between family members.

Parents often observed that the unlimited Internet connectedness caused a pattern of escalating stress to their adolescent, mainly due to the fast-paced, immediate, and persistent nature of SM communication. As one father commented:

… and the thoughts running really quickly … they are trying to keep in touch with 20 people at once … talking to this person, talking to that person … God! You know … one at a time, you know …

Parents expressed their concerns about the addictive patterns of SM use that meant that parents were potentially required to manage a form of addictive behavior, often without proper guidance or strategies.

#### My Child Knows More than Me, I Cannot Keep Up With the Technology

It’s also harder to keep up though, because they have … all this media, I have no idea! I’ve obviously heard about Facebook, Instagram, I don’t really know much about it … (Mother)

Parents reported that their children were far more proficient and knowledgeable in using the Internet and SM, a situation that often led to a role reversal, where children became the experts and parents were the novices. Yet, the children’s technical expertise did not equip them to deal with the emotional consequences of information they were, thereby able to obtain. As a result, parents often felt they had minimal control over the content or people their adolescents were exposed to on the net, but left to deal with any negative emotional consequences. As described by one mother:

You can’t keep informed with something you wouldn’t even think to look into … everything was new for us; it was like; ‘oh my gosh, I didn’t know you could do that – now we know

### Parents’ perceived inability to protect their child

Parents’ perceived inability to protect their child clustered four sub-themes that reflected the outcomes or consequences of SM and the Internet excessive use. They included (a) SM can potentially exacerbate my adolescent’s depression, (b) the abundance of information can be confusing and potentially harmful for my adolescent, (c) Cyberbullying, and (d) SM has changed the meaning of friendship.

#### Social Media can Potentially Exacerbate My Adolescent’s Depression

In many sessions, parents explicitly acknowledged that their concerns regarding their adolescent’s use of SM and the Internet were elevated due to their depressive disorder. As one mother described:

The difference between what she (her adolescent daughter) would look at and a healthy happy adolescent would look at is very different, so my concerns for her are huge, because I really don’t know how far she would go and what she would do, so that has always been a big issue for us …

Other parents described incidents where they believed their child’s mental health deteriorated as a result of interactions on SM. As one mother commented:

and it can take one post that somebody doesn’t like to set them off.

Many parents shared this sentiment, often describing how neutral content on the net, could be interpreted by their depressed adolescent in a negative way, which often led to exacerbation of their depressed mood or significant distress.

#### The Abundance of Information can be Confusing and Potentially Harmful for My Adolescent

Some parents expressed their frustration with the easy access children currently have to a wide variety of content on the net, some of it not age appropriate and this can potentially confuse the adolescent that does not have the experience or the tools to disseminate the content, and fully grasp its meaning. As one mother described:

They’re so much more aware of everything. Things that may be very unhealthy … sometimes I wonder, because of the information about all sorts of things being so accessible, whether they get caught up in the knowledge, and it may be confusing them more …

In answer to the facilitator’s question as to what bothered her specifically, the mother replied:

The labeling … she likes to get a bit of this information and put it onto herself … I just think she’s trying to look for labels for things she didn’t even start to experience yet.

Parents were also concerned about potentially harmful content that could elicit harmful behavior, particularly in the light of their child’s depression, as illustrated by a mother describing how information on the Internet allegedly supported her daughter’s suicide attempt:

[her daughter] had her first suicide attempt … she took a whole lot of panadol and the panadol was going to … that’s what she told me. the panadol makes you, when you hang yourself, it’s not as painful … and I’m thinking; where would she get it from? Obviously from the Internet

Participants mentioned various Internet groups that were harmful in nature, such as suicide groups that provided support and information for people who wanted to commit suicide. Legitimizing harmful acts through online support groups was one of the dangerous effects of SM use that parents felt they had no strategies to deal with effectively, as adolescents were more prone to peers’ influences and less to parents’ guidance.

#### Cyberbullying

The discussion around cyberbullying was strongly tied to the theme of excessive communication and to the pervasive nature of cyberbullying. As one mother described:

When we left school, we LEFT school … no mobile, no computer … if anyone was bullied or upset, it was gone straight after school and we only had to put up with them 6 hours during the day … but now … they wake up to messages, they go to bed with messages …

As evidenced in previous themes, parents often felt that their ability to protect their child from these harmful interactions was greatly reduced due to the virtual and pervasive nature of the Internet. The increased level of exposure through SM meant that the victim was often exposed to ridicule and shame from a much larger group of peers who had access to the harmful content at all times. Furthermore, parents felt that their adolescent’s depression already made them an easy target for bullying, and that their vulnerability exacerbated the harmful effects.

Parents also noted that the anonymity on the Internet, coupled with the lack of immediate consequences to the offender, made bullying more prominent and sinister, as a father explained:

I think the whole generation has lost their ability to read a face because they say what they like and they don’t have consequences … and that causes even more trouble.

#### Social Media has Changed the Meaning of Friendship

The discussion around friendships in the digital age revolved around patterns and quality of friendships. Parents reported that most of their adolescent’s social interactions were done through the net:

They do a lot online. They’ll catch up every few weeks … they spend most of the time Skyping … or Facebook

Friendships in general were characterized as superficial, and reflected the different world view between adolescents, who perceived the virtual world as an extension of the physical world and therefore treated online friendships as real and meaningful, and parents, who perceived the virtual existence as inferior to physical existence, as demonstrated by a mother:

It tended to be: ‘that person, that person … I know them because they’re on Facebook and they are my buddies … ‘that person was really nice to me so that’s my real best friend’ and really know nothing about them or who knows where they came from …

Parents also felt that there was a shift toward preference for quantity over quality, which put extra pressure on their adolescent to be liked by as many people as possible on the net, as one father described:

They are trying to get as many friends on Facebook … and they are trying to get as many ‘likes’ … if they don’t get many ‘likes’ then they are ‘down’ here [points down low], and if they get more ‘likes’ they move up. When we were younger, hopefully you’d get acceptance from your small group, whereas now, kids have such a big group they want to get accepted by everybody!

The cost of superficial friendships, as described by some parents, was tied to harmful online social interactions with peers that were considered friends, which could often be escalated to cyberbullying. In addition, the reality where most social interactions were occurring online meant that parents often felt that they were unable to monitor and protect their adolescent from negative influences and interactions, as they often never met the perpetrators face-to-face.

Parents expressed their concerns that their adolescent’s distorted perception of friendship, in which disrespectful and nasty behaviors were viewed as a part of a normal social exchange, meant that their adolescent did not acknowledge real friendship. As one mother described:

She’s got friends that have been long term family friends, but she says: ‘but they’re not really my friends, they’re more like sisters’. Well, isn’t that one of the best things that you can possibly have?. I think … she doesn’t really know what a good friend is.

Some parents were concerned that this conceptual change in friendship could be particularly damaging for their adolescent, due to their depression.

## Discussion

As a qualitative study, the current findings are based on a relatively small number of parents who provided rich data on their family experiences of parenting an adolescent who presented with a depressive disorder. These data were collected in the context of families who were participants in a clinical trial. The qualitative data therefore are best considered suggestive of possible hypotheses and further lines of research. Two overarching themes were extracted in the thematic analysis. The first was that in relation to their adolescent’s SM and Internet use, parents described a sense of *loss of control over the family environment*. The second theme suggested that parents feel that their adolescent’s use of the Internet detracts from their ability *to protect their adolescent*.

Both themes are consistent with previous research on parent’s perceptions regarding SM and Internet use (5). Previous research has also shown that parents consider that their child is more technologically competent and often more informed about SM (4, 5). Our study adds that, in this clinical context, parent’s authority over their child was greatly compromised (as explored in theme My Child Knows More than Me, I Cannot Keep Up With the Technology). The family routine was also affected since adolescents were often “online,” changing plans and responding to events spontaneously, often disrupting planned family events and creating, at its extreme, a chaotic atmosphere for family life. It should be noted, however, that the nature of the data does not allow us to conclude definitively that adolescent use of the Internet contributes substantially to a lack of family cohesion or that aspects of family dysfunction were already present. However, further studies should investigate this important hypothesis.

These additional stressors appeared to disrupt family cohesion and create new conflicts within the family. Parents consider that the virtual environment requires more intensive parental monitoring. This includes explicit demands to access what the adolescent often perceives to be “private content” or to impose time limits on Internet use. Parents who felt they had lost control also typically felt they had lost the ability to protect their child from harm. In some cases, this perceived lack of control makes the Internet a source of parental fear and high conflict. This was particularly impactful on the parent’s confidence and role definition since it clashed strongly with their view that protection is one of the parent’s primary roles. The theme of parents’ compromised ability to protect their children was perhaps exaggerated due to the clinical nature of the sample whose depressive disorder may render them more susceptible to the influences of the Internet and SM (1–3). In support of such an interpretation, some parents differentiated between their depressed adolescent and non-depressed adolescents in their reaction to online material.

In the current study, many parents did report what they perceived to be harmful effects of SM use on their child’s well-being. This is consistent with previous research that highlighted the relationship between the presence of depression and heavy use of SM and the Internet (1–3). However, given limited numbers and the qualitative method, the current study cannot infer the directionality of this association or if this parental perception was indeed accurate. It is possible that the use of SM and the Internet exacerbated an already existing mental disorder, but was not a contributing *cause* of their adolescent’s depression. Such an assertion would require longitudinal investigation.

Consistent with previous research, parents felt that the anonymous nature of online communication allowed for greater aggression (12, 13). Some research on adolescents’ perceptions suggests that they do not strongly differentiate between virtual and “real-world” friendships, and treat these interchangeably. As such, online interactions may be of greater influence than parents anticipate (10), and suggests that some parental attitudes may lag behind new social trends.

In the current study, parents reported a lack of strategies that would help them to better manage their relationship with their adolescent in the digital age and lack of clarity as to how to assume a parental role in an online environment. Many parents felt isolated and unsupported, left to deal with the consequences of the negative impact of the use of SM and the Internet on their adolescent. There is clearly a role for the further development and evaluation of educational and therapeutic resources to support parents in this task.

There is also a need to develop and evaluate valid self-report measures to capture parent’s attitudes toward and behaviors in relation to their children’s SM and Internet use. Once incorporated into larger population studies such measures could also examine associations between these parental attitudes to SM and family demographic factors, such as age, education, and gender. It is notable that in the current study its method, sample size, and the requirements of confidentiality prevent us from reporting such associations.

This study is qualitative, descriptive, and based on the views of small number of parents but it does provide a snapshot into the family dynamics of depressed adolescents and their interactions with SM. It also provides a vivid representation of the influence of SM on family functioning in this context. Further research is needed to confirm whether there is a causal relationship between excessive use of SM and the Internet and adolescent depression.

## Author Contributions

AL – is a senior Clinical Psychologist, supervisor, co-developer of the BEST-Mood intervention, and was the senior Trial Manager on the project. TK – is a supervisor and assisted in co-developing the intervention. Both AL and TK were involved in data analysis. GG – completed an Honors dissertation on the topic and was involved in analyzing the qualitative data and reviewing literature, MB – worked as a research assistant on the project and prepared the current paper for submission, LP – worked as research assistant on the project and assisted in the review of the current paper, CJ – worked as a research assistant on the project and assisted in transcribing the recordings for analysis.

This study was carried out in accordance with the recommendations of the Deakin University Human Ethics Research Committee with written informed consent from all subjects. All subjects gave written informed consent in accordance with the Declaration of Helsinki.

## Conflict of Interest Statement

The authors declare that they have no competing interests. The authors hold intellectual property responsibility for the interventions under study.

## Funding

We are also grateful to the following organizations and funding bodies who have financially supported this clinical trial: Australian Research Council, beyondblue: The National Depression Initiative, Drummond Street Services and Stepfamilies Australia, Australian Drug Foundation, and Deakin University Center for Mental Health and Wellbeing. Our major funding source (the Australian Research Council: LP110200167) had no role in the design of this study and will not have any role during its execution, analyses, interpretation of the data, or decision to submit results. Our industry partners have provided lesser financial and in-kind support, and have been involved in assisting its execution according to the trial protocol (beyondblue: The National Depression Initiative, Drummond Street Services and Stepfamilies Australia, Australian Drug Foundation, and Deakin University Center for Mental Health and Wellbeing).
